# Establishing the distribution of *Carpophilus truncatus* in Australia using an integrative approach for an emerging global pest

**DOI:** 10.1038/s41598-024-70687-x

**Published:** 2024-08-22

**Authors:** Stephen James Tobin, John Paul Cunningham

**Affiliations:** 1 Agriculture Victoria Research, Agribio Centre for AgriBiosciences, 5 Ring Road, Bundoora, 3083 Australia; 2 School of Applied Systems Biology, La Trobe University, Melbourne, 3086 Australia

**Keywords:** Pest invertebrate, Integrated pest management, IPM, Species distribution modelling, SDM, Insect ecology, Ecological modelling, Invasive species, Ecology, Agroecology

## Abstract

The nitidulid beetle *Carpophilus truncatus* is rapidly becoming a major pest of nut crops around the world. This insect first infested Australian almonds in 2013 and has since escalated to be the preeminent insect pest for the industry. Data pertaining to *C. truncatus* distribution are scant, but without awareness of its origin, distribution, and ecological factors that influence distribution, efforts to understand and manage the insect as a pest are stymied. Here, we employ an integrative approach to gain a multifaceted understanding of the distribution of *C. truncatus* in Australia. Methods employed were (1) reviewing historical records in insect collections to establish the presence of *C. truncatus* prior to commercial almond horticulture, (2) field trapping of insects to establish presence in regions of interest, (3) laboratory trials to determine the thermal limits of the organism, and (4) correlative species distribution modelling to describe its current distribution. We find that *C. truncatus* is more widespread across Australia than was previously known, with historical records preceding commercial almond production in Australia by a century. The methods developed in this study can be applied elsewhere in the world where *C. truncatus* is an emerging pest, or to novel pest species as they arise with increasing frequency in a globalised and warming world.

## Introduction

The almond carpophilus beetle, *Carpophilus truncatus,* recently emerged from relative obscurity to become a major problem for nut growers in several regions of the world^1^. Previously unknown in Australia, larval and adult *C. truncatus* were first observed infesting almond kernels in 2013, as many large orchards began to reach maturity^2,3^. Direct damage caused by the beetle grew to an estimated cost of $12 million AUD in just three years^4^, while the identity of the insect remained unconfirmed until 2020 when it was described in the literature as *C. truncatus*^3,5^. A similar pattern of crop damage emerged in the Catamarca region of Argentina, where an established but expanding walnut industry faced huge post-harvest losses from a *Carpophilus* beetle that was found to be a genetic match with *C. truncatus* (at that time referred to as *C.* nr. *dimidiatus*) in Australia^3,6^. Italy too, as of 2019, faces the burgeoning problem of walnut post-harvest damage from *C. truncatus*, and the spread of the insect has been monitored in walnut orchards and storage facilities throughout the Campania region^1^. Then in 2023, *C. truncatus* was identified damaging almonds and pistachios in the San Joaquin Valley in the United States of America^7^, where 78% of the world’s almonds are produced^8^ and where its potential impact on nut industries is uncharted.

The origins and distribution of *C. truncatus* are shrouded by a fragmented history of taxonomic revisions and identification difficulties^3^. Understanding where this insect has come from, and the environments it inhabits beyond almonds is, however, essential for disentangling its ecology and enabling management of the insect as an important agricultural pest^9^. Obtaining distribution data for a little-known insect species such as *C. truncatus* can be challenging*,* with different methods having inherent merits and pitfalls. A thorough search of scientific literature, grey literature, and database observations of the species is a natural starting point^10,11^, but online records can be insufficient in such cases. Accessing specimens from curated insect collections can also provide valuable historical records of a species^12^, but *C. truncatus* has historically proven to be an elusive insect, so it may not be well-represented in collections^13^. Accurate documentation of *C. truncatus* within collections is also hampered by the fact that the insect is difficult to distinguish morphologically from sibling species within the *Myothorax* subgenus^3^. Moreover, records are liable to geographic biases or may contain insufficient geographic information^14^. Field work to obtain new specimens may yield presence or absence records across areas of interest^15^, with methods ranging from targeted manual searches to surveillance trapping with specialised equipment. Trapping and collecting exercises are, however, typically expensive and time-consuming endeavours, and the effectiveness of trapping will be limited by if, when, where and how the species can be effectively trapped^16^. A recent boon for exploring the origin and distribution of *C. truncatus* is that an effective lure comprising the beetles’ aggregation pheromone and host volatiles has now been developed for the species^17–19^. This lure can be used to trap and monitor the insect in orchard and non-orchard environments, providing insight into suitable habitats and supplying sufficient presence-absence data for construction of correlative species distribution models (SDMs).

Aside from direct evidence from collected specimens, physiology-based predictive models can help ecologists understand the wider distribution of a species^20^. Laboratory experiments are designed to collect physiological data such as the thermal envelope or humidity preferences of an organism, which are then compared to climate data to estimate suitable geographic space for survival and growth^21–23^. Advantages of physiological approaches include the ability to forecast distributions into the future and extrapolate into new geographic regions^24–26^. Some limitations of physiology-based approaches are that they do not account for biotic factors that influence distribution (such as mutualisms, predator–prey or competitive interactions), nor do they consider ongoing evolution and adaptation by species^27–29^. Full mechanistic SDMs are laborious to produce, requiring complete data sets and a thorough knowledge of ecological parameters^30,31^. Even so, physiological variables can be useful for understanding invertebrate ecology^32^ and species distributions^33,34^ per se.

In this study, we used an integrative approach that combines historical records with targeted field trapping and predictive modelling to explore the distribution of *C. truncatus* in Australia. Using several complementary methods allows a multifaceted understanding of distribution while mitigating the limitations of any single approach. First, this study aimed to determine if *C. truncatus* was naturalised in Australia before the almond industry became established, or if the pest is more likely to be a recent incursion from another part of the world. This was achieved by investigating literature, online data sources and insect collections to find records of *C. truncatus* preceding its appearance in Australian almonds. Second, this study aimed to uncover the presence or absence of *C. truncatus* in areas of interest that are geographically distant from almonds using traps with a species-specific lure. Third, this study aimed to establish how widespread the insect could become in Australia by determining the relative thermal suitability of the Australian continent for *C. truncatus*. Laboratory experiments measured development and survival of different insect life stages across a range of temperatures. Development data were then compared to hourly predicted soil temperatures across Australia to estimate the number of contiguous generations of *C. truncatus* that could develop over a year, bounded by the critical thermal minimum (CTmin) and critical thermal maximum (CTmax) of the hardiest life stage (adults). Fourth, this study estimated the current realised distribution of *C. truncatus* in Victoria, the state in Australia where almond production is most concentrated and where the pest is prevalent. This was achieved by systematic trapping of the insect with lure-based traps and correlative SDM.

## Methods

### Historical Australian records of *C. truncatus*

A search of available online literature and database records was conducted to uncover documentation on *C. truncatus*; in particular location-based records for the insect in Australia, as well as records of habitat preferences, host associations or ecology. The academic search engines Google Scholar, Semantic Scholar, Science.gov, and Trove: National Library of Australia were used to find journal articles, reports or other records of *C. truncatus*. Atlas of Living Australia, GBIF, and iNaturalist databases were explored to uncover additional online distribution records. References of returned literature were also reviewed for additional information or records.

Insect collections near the east coast of Queensland, over 1000 km north of Victoria, were visited to uncover any historical *C. truncatus* specimens in tropical or subtropical regions, as well as collections nearest to almond production regions. Agricultural and biosecurity collections were a focus, based on the high representation of *Carpophilus* species in agricultural settings^35,36^. All *Carpophilus* and *Urophorus* specimens from the Queensland Primary Industry Insect Collection (QPIIC) were examined under compound microscope to visually assess external morphological features and assess taxonomic designation. Due to the high number of *Carpophilus* specimens available at the Victorian Agricultural Insect Collection (VAIC), only databased adult specimens with known host associations or specimens from non-almond orchard locations were examined. Other insect collections visited include the agricultural insect collection at the Queensland Department of Primary Industries in Mareeba and the insect collection at the Daintree Research Observatory at Cape Tribulation, Queensland. In addition, images of *Carpophilus* specimens were obtained from the NSW Biosecurity Collection (ASCT) in Orange.

### Lure-based trapping

A trapping regimen was established to determine whether *C. truncatus* can be found in native bushland in Victoria. A total of 80 pheromone traps were placed in regional, state and national parks for six weeks during either the 2021–22 or 2022–23 Australian summer and early autumn (December–March). These trapping times align with *C. truncatus* movement and research activities in almond orchards in Australia^2,4^. Trapping locations were identified through consultation with local state government staff in order to encompass diverse habitats and to ensure trapping covered ecological gradients^37^.

*Carpophilus* traps consisted of an enclosed black bucket trap (Catcha System, Bugs for Bugs) secured in place with a steel picket and ring with straps. Traps contained a pheromone lure made up of the insect’s aggregation pheromone and a separate liquid semiochemical blend (Lure AI) made up of fermentation volatiles, which works synergistically with the pheromone to increase attraction^17–19^. Approximately 200 mL of the Lure AI was poured into a plastic jar and the mouth of the jar covered with mesh to exclude insects while allowing release of volatile organic compounds (VOCs). Due to the volatility of Lure AI, replacement was necessary every two weeks. A 1.5 × 1.5 cm insecticide block (active ingredient, Dichlorovos) was placed in each trap to kill insects upon entering. The trap opening was covered by a wire mesh that allowed the passage of *Carpophilus* while precluding larger invertebrates. Dead insects were collected from the traps every two weeks. Trap samples were dried if necessary and kept frozen until specimens could be morphologically identified in the laboratory under a dissecting microscope. The identity of ambiguous specimens thought to be *C. truncatus* was elucidated by extracting DNA using a Qiagen DNeasy Blood and Tissue Kit as per Martoni et al*.*^38^, with COI barcode regions amplified by PCR and amplicons sequenced by Macrogen Inc (Seoul, South Korea).

For the first season of trapping, naturally produced *C. truncatus* pheromone was used in traps, extracted from male insects feeding on almonds. Pheromone and almond host volatiles were harvested in the laboratory by dynamic headspace sampling of VOCs from 200 to 800 males feeding inside a glass collection vessel. VOCs were harvested continuously from six collection vessels over a 12-week period. Males were fed on almond kernels (only males produce the pheromone when provided a food substrate^17,18^), and VOCs were collected using solid phase (Porapak Q) filters. Filters were replaced weekly and VOCs eluted with hexane. The pheromone was not further isolated from almond volatiles as these host (and fermentation) volatiles are known to attract *Carpophilus* species*,* including *C. truncatus*^4,39^. The VOCs in hexane were then loaded on a compact cotton swab with 50 μg of vegetable oil and encased in a 1 mL micropipette tip sealed with an aluminium crimp cap. The release of pheromone from these devices was confirmed by gas chromatography-mass spectrometry, with detectable quantities released for at least one week under laboratory conditions. Lures were made 1–3 days before being placed in the field and were kept on ice until they were deployed. By the second season of trapping, the molecular structure of the pheromone had been determined and a synthetic version produced^17,18^, and this was loaded onto rubber septa instead of the lab-collected pheromone as per Bartelt and Hossain^39^. The synergistic Lure AI blend was prepared and deployed in traps as per the specifications of the patent^19^ for both seasons of trapping.

In addition to the systematic trapping regimen for Victoria, trapping was performed for 1–2 weeks in areas around Brisbane and tropical Far North Queensland (FNQ). The recorded distribution of *C. truncatus* includes tropical islands in the Indian and Pacific Oceans, and the insect has been recorded on the fruits of *Hernandia sonora*^40,41^, a plant that inhabits the east coast of Queensland. The three regions that were selected for trapping in Australia (Victoria; Brisbane Metropolitan Area and Lamington National Park; FNQ) represent distinct climatic zones spanning the eastern side of continental Australia, covering the tropical far north, temperate far south, and the subtropical mid-point of the two. Trapping in Queensland was conducted in March of 2023 utilising the trap specifications detailed for the second season in Victoria.

### Physiological data

Temperature response trials were conducted to investigate *C. truncatus* development and survival across their full range of habitable temperatures. Twenty-four insects of each discrete life stage (egg, larva, pupa, adult) were individually placed in separate 200 μL dome cap 8-strip tubes and incubated at constant temperature in a thermocycler^42^, recording development time and survival for each insect. Development and survival of *C. truncatus* were tested at 4 °C increments using a fresh cohort of insects for each temperature, until the CTmin and CTmax were determined. Larvae and adults were placed on an artificial feeding substrate made of soybean meal and sugar, while eggs and pupae were placed on moistened sterile cotton wool or very fine vermiculite, respectively. Insects were sprayed with water every second day or as required at hotter temperatures to ensure adequate humidity was maintained within incubation tubes. The temperature of the incubator was routinely verified using a probe thermometer. Adult insects were incubated for a maximum of two weeks, with any deaths recorded daily. Of the 24 replicates at each temperature for the adult life stage, 12 freshly-eclosed males and 12 freshly-eclosed females were used, while for egg and juvenile life stages individuals were chosen at random, all from a laboratory-reared colony with an approximately 1:1 sex ratio.

The temperature response data were subsequently used to predict the number of generations *C. truncatus* can theoretically complete in a year across Australia, using methods described in Kearney^34^. In short, a linear model is used to fit temperature to development rate (eggs, larvae and pupae inclusive) over the range of relevant biological temperatures. For *C. truncatus* the temperature range used was 14–34 °C, with 38 °C excluded because few insects survived to complete development at this temperature for both larvae and pupae (3 and 2 insects out of 24 respectively). Cumulative development of contiguous generations was calculated for the full year by applying the linear model to grid-format microclimate data. Each freshly-eclosed adult generation was assumed to be capable of reproduction immediately. The microclimate data used was simulated MicroclimOz^34^ hourly temperature data for soil across Australia at 2.5 cm depth in 2017, the most recent year available. An output raster was generated depicting the number of *C. truncatus* generations that could theoretically be produced across Australia in 2017. A minimum temperature mask was produced using the July (winter) mean minimum temperature layer provided by the Australian Bureau of Meteorology^43^. Locations where the July minimum temperature average was below the survivable temperature threshold for the hardiest life stages—eggs and adult beetles (2 °C)—were masked in blue. Neither the maximum survival temperature for eggs (42 °C) nor adults (46 °C) was exceeded by the maximum average temperature for December (summer), so no maximum temperature mask was produced.

### Correlative species distribution model

A presence-absence SDM was used to predict likelihood of *C. truncatus* presence across Victoria based on the results of lure-based trapping in the state. WorldClim climate surfaces^44^ were used as predictor variables for a generalized linear model (GLM) with presence-absence as the dependent variable and a logit link function. Predictor variables were rescaled so that each variable had a mean of zero and standard deviation of one as a means of standardising the predictors, and predictor grids were resampled to ≈ 10 km resolution to lessen the risk of model overfitting and reduce computational demand. Correlations between predictors were used to narrow down the number of WorldClim layers used in candidate models by iteratively removing the most correlated predictors while attempting to retain variables that were useful in similar models in the literature^45–47^. Predictor values were then extracted for trap locations and candidate models produced. Twenty-two models were built using different combinations of the refined predictor variables. Candidate models were then compared using AIC values and the best model with the lowest AIC score was retained for output predictions. The final model was applied to the predictor variables over the area of interest to produce an output raster estimating the likelihood of *C. truncatus* presence across Victoria.

## Results

### Historical Australian records of *C. truncatus*

**Fig. 1:**
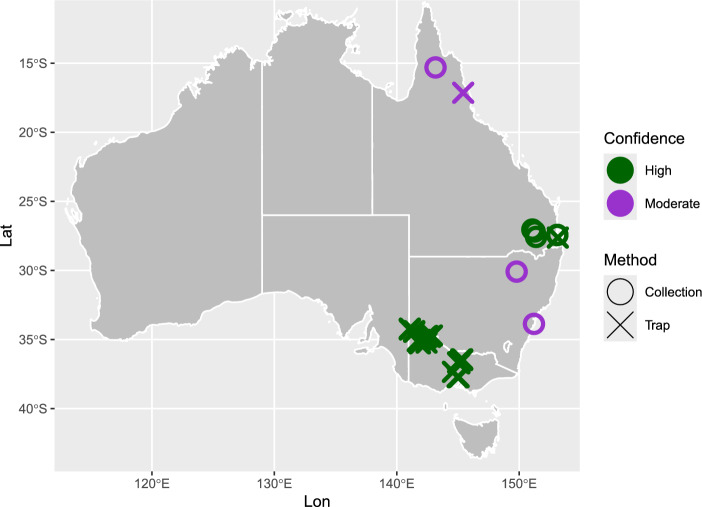
*C. truncatus* specimens trapped or assessed during this study. Crosses indicate specimens obtained from traps during field work; open circles indicate existing insect collection specimens that were newly re-identified as *C. truncatus* in this study. Confidence in *C. truncatus* identification: green symbols = high confidence (clear morphological characteristics or DNA sequencing); purple symbols = moderate confidence (putatively *C. truncatus* but possibly *C. imitatus*^3^).

Agricultural and biosecurity collections across the eastern states of Australia were investigated to uncover historical occurrences of *C. truncatus* (Fig. 1). Most notable among the historical specimens were *C. truncatus* with collection dates from 1905 to 1962 identified among the QPIIC collection from five different locations near Brisbane. Also from QPIIC was a specimen possibly from Dixie in FNQ dated 1962, but unfortunately the location was not confidently attributed on the determinavit slip (collection label). Images of specimens from the NSW collection showed the distinctive characteristic of tibial constriction for male specimens^3^, but *C. truncatus* could not be confidently distinguished from the morphologically similar *C. imitatus*. No collection specimen labels contained information pertaining to host plants.

No historical records of *C. truncatus* outside of almond and pistachio orchards were found for Australia in any of the online databases searched, and from the scientific literature only three detections of *C. truncatus* DNA in fruit fly traps near Shepparton were discovered within the supplementary material of Piper et al*.*^49^.

### Lure-based trapping

Specimens of *C. truncatus* were identified from trapping along the east coast of Australia. Trapping in Victoria resulted in 190 individual *C. truncatus* adults collected from bushland habitat, with many specimens very distant (> 200 km) from recognised almond growing regions, and as far south as the Macedon Ranges and the northern fringe of Melbourne. The species was also found in traps set up in Queensland, and although few specimens were collected one specimen from Daisy Hill near Brisbane was confidently identified as *C. truncatus*, confirmed by molecular analysis. Three female specimens caught in the FNQ traps near Walkamin were determined to be *C. truncatus* based on morphology, but molecular analysis was unable to confirm morphological identification as DNA barcoding was unsuccessful. One specimen in particular, at 2.8 mm in length, accords with *C. truncatus* but not the smaller *C. imitatus*, which is described with a maximum length of 2.4 mm^3^. Genetic sequences produced in this study can be accessed via the GenBank® genetic sequence database, see accession numbers PP645855 to PP645861.

In addition to *C. truncat*us, other nitidulid beetles were also recovered from the pheromone traps. The cosmopolitan detritivore *C. hemipterus* was found in many traps, especially in fruit-growing regions, with 987 *C. hemipterus* recovered from a single trap in Annuello Flora and Fauna Reserve in northwestern Victoria. *Brachypeplus* spp. were found over much of the trapping range, including FNQ and southern Victoria, although only a few individuals were usually recorded in any given trap. *Urophorus humeralis* specimens were also recovered from Victoria through to FNQ.

### Physiological data

**Fig. 2:**
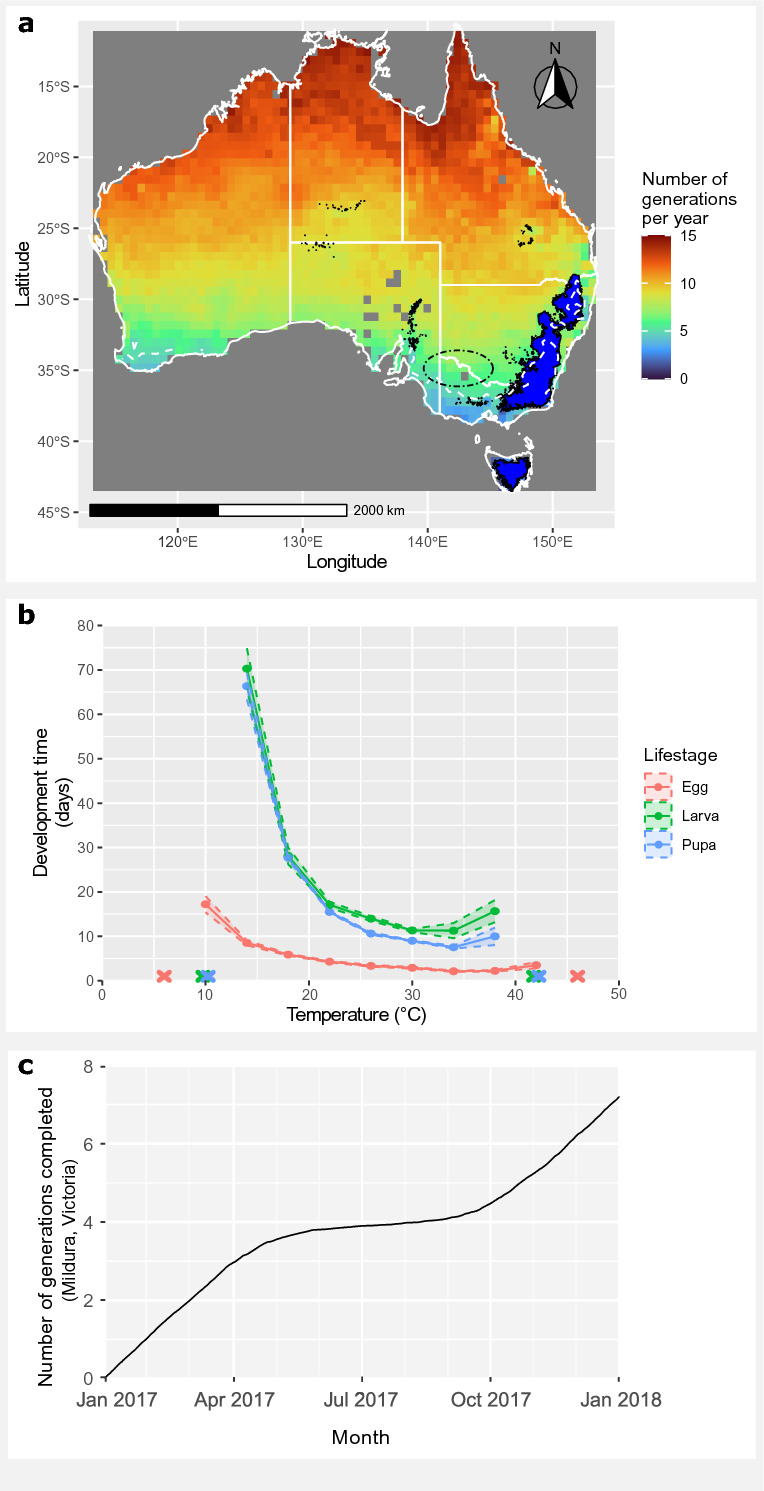
Temperature-dependent development of *C. truncatus* in Australia. a. Australia-wide map projection depicting the theoretical number of contiguous generations *C. truncatus* can complete in a year based on temperature-dependent development rates. The blue mask (black outline) covers areas where the minimum temperature is predicted to be too cold for adult *C. truncatus* to survive according to the temperature-performance experiments. The broken white line depicts the contour where the theoretical number of generations completed = 5. The black dot-dash ellipse encompasses the main commercial nut growing regions in Australia. b. The number of days for *C. truncatus* developmental life stages (eggs, larvae, and pupae) to complete development over the full range of survivable temperatures. Crosses indicate that insects were incubated at the temperature, but no individuals survived to complete the life stage (n = 24 per life stage, per temperature). Larva and pupa crosses are jittered slightly so that overlapping points can be seen. Dotted lines represent 95% confidence intervals. c. Cumulative number of generations predicted to reach developmental completion for Mildura in the major almond growing region of Victoria, over a 12-month period based on the temperature-development linear model. We used the most recent year of simulated soil temperature data available, running from 1st Jan 2017 to 1st Jan 2018^34^.

The CTmin and CTmax for *C. truncatus* larvae and pupae are 10 °C and 42 °C respectively, making these the most thermally restricted life stages, while egg CTmin and CTmax were 6 °C and 46 °C respectively (Fig. 2b). Development was much slower at cooler temperatures for every developmental life stage, and at the extreme high temperatures development rate also showed a small decrease before survival ceased at CTmax. Adult insects could survive two weeks of incubation at temperatures between 6 °C and 46 °C, though a lower proportion survived the full 2 weeks towards the extremes of their thermal tolerance. In Mildura within the Sunraysia almond growing region, *C. truncatus* development was calculated to exceed seven generations in the 2017 calendar year, with development considerably slower from May to October compared with the rest of the year (Fig. 2c). Refer to supplementary datasets S1 and S2 online for adult survival or egg and juvenile development data respectively.

Based on the average rate of development, the maximum number of theoretical contiguous generations for *C. truncatus* in Australia was 14, evident along the northern part of Australia where soil temperatures are typically warmer according to the MicroclimOz soil layer (Fig. 2a). The number of theoretical generations approaches zero towards the southern parts of Australia. A cold mask covers areas where the most cold-resilient life stages of *C. truncatus*—eggs and adults—are unlikely to survive the average ambient minimum temperature of July. This mask covers the eastern parts of Victoria and NSW, as well as Tasmania. The five-generation contour also covers a similar extent of southeastern Australia, as well as the southernmost part of southern Western Australia. Refer to supplementary dataset S3 online for the summarised temperature development data used to generate this model.

### Species distribution model

**Fig. 3:**
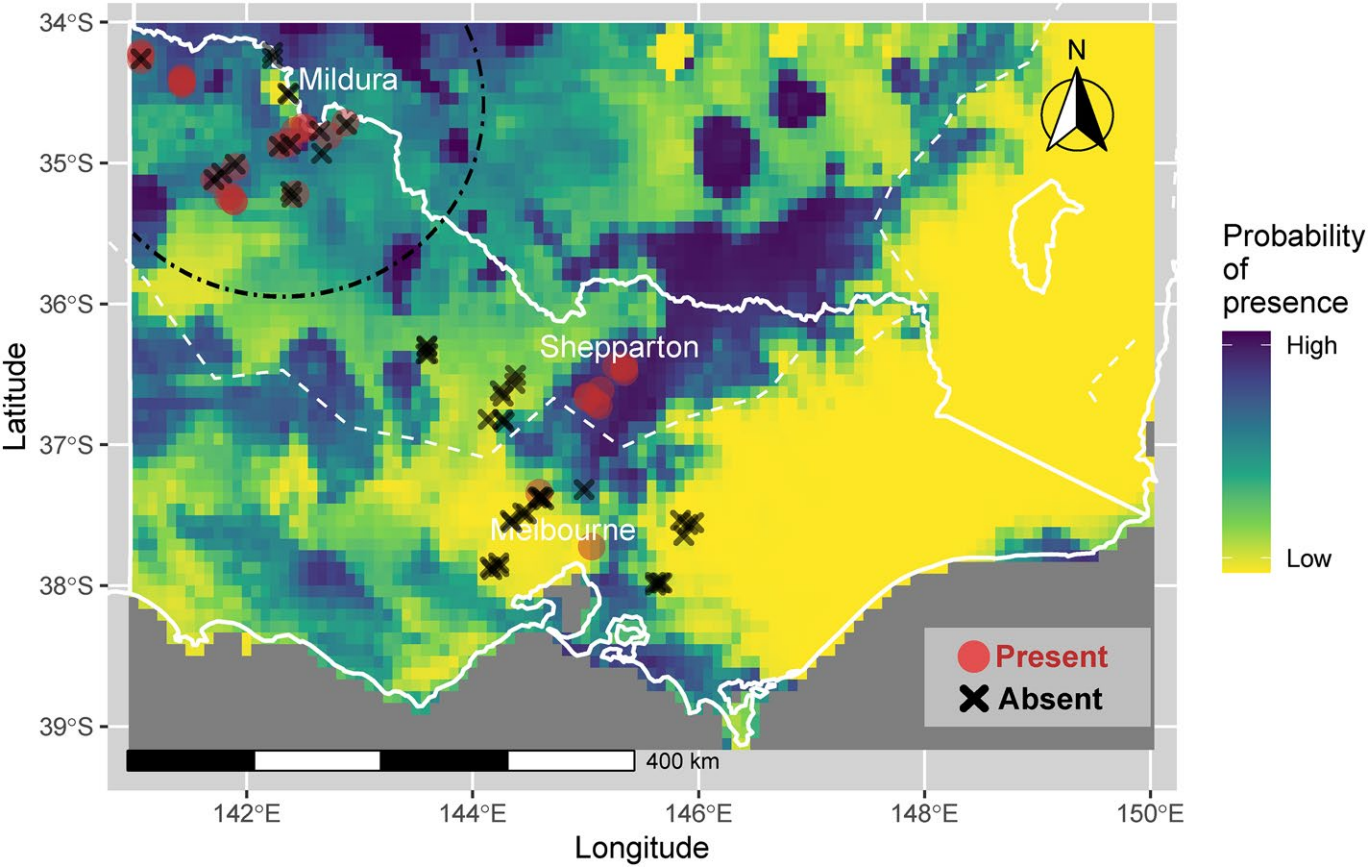
Species distribution model output indicating the likelihood of *C. truncatus* presence across Victoria. Dark blue represents high likelihood of presence, light yellow indicates low likelihood of presence. The white dotted line is a contour indicating five complete generations according to the temperature development prediction, copied from the contour in Fig. 2a. The black dot-dash arc encompasses the area with the highest density of large almond orchards in Australia, a growing region where *C. truncatus* is the predominate almond pest. Trap locations, used for model building, are depicted as red presence dots (traps that captured *C. truncatus*) or black absence crosses (traps that did not capture C. truncatus), displayed at 50% opacity to better visualise overlaid points (areas of high trap density). City names are approximate locations to avoid obscuring data.

The likelihood of *C. truncatus* presence varies over the state of Victoria according to the SDM output (Fig. 3). In the Sunraysia growing region towards the northwest of the state, there is an intermediate-to-high probability of a capturing *C. truncatus* under the same conditions that the training data were collected. Near the Shepparton region, where all traps placed captured *C. truncatus*, the predicted probability of presence approaches one. Further south, and particularly towards the colder climes of the southwest, the probability of presence predicted by the model is mostly zero. In this area, the five-generation contour produced using the temperature-development data aligns with a low likelihood of *C. truncatus* presence predicted by the SDM.

After reducing the number of predictors of interest based on correlation and literature findings of previous SDMs modelling other invertebrate species, the variables retained for the final model were Bio4 (temperature seasonality), Bio6 (minimum temperature of coldest month), Bio7 (temperature annual range), Bio8 (mean temperature of wettest quarter) and Bio15 (precipitation seasonality). The model that included all five of these variables returned the lowest AIC value compared to models with only subsets of these predictors. Of the retained variables, Bio4 and Bio6 were ranked the most valuable in terms of cumulative deviance explained (see supplementary material S5). This is congruent with the findings of the physiology-based model indicating minimum temperature is a limiting factor for the distribution of *C. truncatus*.

The area under the curve value (AUC) score of 0.88 for the receiver operating characteristic curve (ROC) indicates good model performance. An AUC score of 0.5 indicates a model has no ability to discern between groups, and scores nearer to 1 approach perfect model performance. Tjur’s R^2^ calculated for the model is 0.42, indicating a moderate strength of relationship. Refer to supplementary dataset S4 online for presence-absence data used to generate this SDM and supplementary notes S5 for further discussion of modelling methods, evaluation and discussion.

## Discussion

Before *C. truncatus* was identified attacking almond crops in Australia in 2013 it was a little known and poorly described insect that was not considered a pest. Now, *C. truncatus* is the foremost pest of almonds in Australia and is rapidly gaining status as a worldwide pest of nut crops^1,6,7^. Our integrative study provides definitive evidence that *C. truncatus* is more widespread across Australia than previously identified^1,3^, with a distribution that includes not only the temperate south, but regions much further north in sub-tropical and tropical states of eastern Australia. Moreover, our study reveals for the first time that *C. truncatus* inhabits environments outside commercial nut orchards based on contemporary trapping and historical collection specimens that pre-date and are distant from established growing regions. The appearance and rapid escalation of *C. truncatus* in almond growing regions in Australia is therefore unlikely to be a result of a recent exotic incursion. Instead, the insect appears to have gone unreported in Australia for many decades, particularly in tropical and subtropical Queensland, and only recently started attacking almond monocultures in the temperate southeast as a booming almond industry reached production maturity.

Historical specimens and trapping show that *C. truncatus* is neither a recent exotic incursion, nor is it restricted to areas where commercial nut crops are grown in Australia. The earliest physical specimen provides evidence that *C. truncatus* was present in subtropical Queensland over 100 years before the insect rose to prominence as a pest of almonds in temperate southern Australian orchards^2,3^. Other re-identified *C. truncatus* collection specimens provide evidence of the insect in Queensland and New South Wales throughout the twentieth century. Many of these trapped and re-identified *C. truncatus* collection specimens are geographically distant from commercial almond orchards, most notably in the state of Queensland, where no known nut crop hosts (almonds, walnuts, pistachios) are farmed commercially. Two female specimens caught in our traps in tropical Mareeba in Far North Queensland are a morphological match for *C. truncatus*, but possibly due to the warm conditions degrading the insect DNA quickly we were unable to extract useable material for DNA barcoding. A *C. truncatus* adult was also trapped near subtropical Brisbane during this study, confirmed by DNA barcode using the COI region.

A formerly non-pest insect such as *C. truncatus* can acquire pest status when the insect encounters a novel host, which can arise when new crops are grown in locations where an insect species is already present, or when it encounters the new host crop upon range expansion^50,51^. While both scenarios are feasible for *C. truncatus* in Australia, it is probable that the insect was already subsisting in Victoria before the introduction of almond monocultures and subsequently made the transition to almonds when the crop was introduced, rather than *C. truncatus* migrating south to Victoria and South Australia coincidental with large orchards in those states massively expanding their production. Further genetic studies investigating the population structure of *C. truncatus* could help resolve the invasion history of the insect^1^. Though physical specimens were only evaluated from the eastern states of Australia, the temperature-development data suggests other states and territories could provide an equally suitable climate for *C. truncatus*. Given the limited extent of nut cropping in Western Australia and the Northern Territory, and the unique habitats available in these regions of Australia, further trapping in these states using available tools would provide interesting comparison to eastern Australia.

More systematic trapping in Victoria consistently found *C. truncatus* over 200 kms distant from almond orchards, implying that this species is also naturalised in temperate regions of eastern Australia alongside almond production regions. The insect was trapped more consistently and in higher abundance in bushland near Shepparton in northern Victoria than bushland near the almond production region northwest towards Mildura, which lies close to the border with South Australia. Thus for *C. truncatus* populations in Victorian bushland habitats, higher abundance of the insect does not appear to correlate with proximity to almond monocultures, meaning almonds are unlikely to be the primary source for the bushland populations and that bushland populations are self-sustaining. It should be noted that different pheromone lures were available between seasons, which may also have some impact on the number of beetles caught between the two regions. On the other hand, migration from bushland is unlikely to have an impact on populations in orchards where the insect is already established, because almond orchards report trap catches orders of magnitude greater than bushland traps^4^. Still, areas where new nut crops are being planted should consider the existence of background insect populations, as predicted by the modelling in this study. New orchard locations within the Goulburn Valley agriculture region around Shepparton, for example, should expect *C. truncatus* to emerge as a pest as orchards mature, and should design orchards accounting for necessary management of the pest. As lucrative nut industries continue to expand production around the world^52^, other countries could utilise the approaches described in this study to establish the distribution of *C. truncatus* for similar purposes.

Very few *C. truncatus* specimens were trapped in Queensland compared to Victoria, despite trapping occurring at the same time of year. The ecological drivers of population abundance for *C. truncatus* in bushland habitats are unknown, but are likely tied to host availability^53^. This insect may have evolved seasonal phenology to synchronise its populations with hosts belonging to its original geographical range rather than the current observed range, as has been proposed for Queensland fruit fly, a notorious native pest in Australia^54^. It may therefore be difficult to predict seasonal abundance and behaviour patterns without further knowledge of the original hosts of the insect. Given that the *C. truncatus* type specimen is from Madagascar, with a distribution including Réunion^40^ and Pacific islands^41^, the subtropical and tropical conditions in Queensland may be more representative of the original range of the insect than what is observed in Victoria. Fewer insects in Queensland traps may be indicative of typical ancestral population dynamics for *C. truncatus*, where the insect maintains low background abundance or a patchy metapopulation but can produce high quantities of offspring when ephemeral resources become available. Alternatively, in temperate environments *C. truncatus* may have escaped from competitive or predator–prey interactions that exist in its native environment^55^, allowing populations to achieve greater abundance in these conditions, which would subsequently be reflected in trap catches.

Evidence of the widespread distribution of *C. truncatus* across eastern Australia implies there are other hosts for this insect among the Australian flora additional to commercial nut crops. In this study, *C. truncatus* was trapped in several regions where no known hosts are present, most notably in bushland habitats distant from cropping regions. Subsequent searches failed to find any live specimens inhabiting these locations, so potential hosts remain unknown. Determining the broader host repertoire of *C. truncatus*, including non-crop hosts, is important for understanding its distribution, ecology, and its potential as a pest of other crops or native plants^56^. To date, the literature records *C. truncatus* feeding on a limited range of nuts^3^ as well as the dried fruit of *Hernandia sonora*^40,57^. Leschen and Marris^58^ and Brown^41^ provide more extensive lists of plant associations for *C. truncatus* (including coconut, coffee, fruit, grains and bread products), though none of these commodities have been formally tested as hosts. To investigate the host range of *C. truncatus*, classical laboratory no-choice trials could be conducted to establish fundamental host range^59^. Metabarcoding plant DNA derived from the gut of insects is also a lucrative avenue for investigating the realised hosts of the insect in non-orchard environments^60^. Ultimately, direct evidence from the field would be required to prove host use in an ecological sense^61^.

Seasonal low temperature appears to be a limiting factor for the distribution of *C. truncatus* in the southeast part of its range in Australia^62^. According to the physiology-based approach, seasonal cold is predicted to restrict *C. truncatus* in the southern part of its geographic range. Correlative SDM found the predictor variables most important for describing the distribution of *C. truncatus* were *temperature seasonality* and *minimum temperature of coldest month*, corroborating the physiology-based approach. Here, the combined insights of multiple approaches demonstrate the advantage of considering distribution not just from the perspective of statistical description, but from an integrative ecological perspective. Given its ecological importance for *C. truncatus*, cold temperature could be leveraged in integrated pest management practices to help manage the species^63,64^. For example, a day-degree tool for predicting insect population growth based biological parameters and local weather station data could further refine the timing of orchard management practices. Such a tool has been developed to predict the emergence of citrus gall wasp (*Bruchophagus fellis*) in southeastern Australia to assist management of the pest^65^. Pest management practices such as hygiene sweeps, chemical spraying and pest monitoring in almonds could be upregulated when the pest is predicted to be released from the population-level suppression it experiences at cold temperatures. For *C. truncatus*, there is also the potential to chill treat stored product to kill insects within almonds using knowledge of insect CTmin^63,66^. Insects used in laboratory trials in this study were exposed to constant temperature, but a factor that might affect *C. truncatus* thermal tolerance in nature is variable temperatures, which can enhance thermal tolerance at hot or cold extremes^67^—future trials should investigate the effect of variable temperature and gradual temperature ramping on the thermal plasticity of this insect.

Given the widespread distribution of *C. truncatus* in Australia, from temperate to tropical climatic zones, it is likely that the insect can inhabit most climates around the world where nuts are produced. The swift manifestation of *C. truncatus* among many of the world’s top nut producing countries including Australia, Italy, Argentina and now USA, means that the insect has already bypassed substantial global biosecurity barriers and may have done so even before it was recognised as a pest. This study has established a more holistic understanding of the distribution of *C. truncatus* in Australia using methods that could be applied elsewhere in the world where this beetle is already present, or where an incursion may pose a serious threat to nut production. Beyond this species, novel pests are arising increasingly frequently^68^ and integrative approaches such as described in this study could be used to better understand pest origins, distribution and incursion risk to benefit global food security.

### Supplementary Information

Supplementary Information 1.

Supplementary Information 2.

Supplementary Information 3.

Supplementary Information 4.

Supplementary Information 5.

## Data Availability

Data generated or analysed during this study are included in this published article (and its Supplementary Information files) or the repositories below: Australian Bureau of Meteorology repository, http://www.bom.gov.au/climate/maps/averages/temperature/. MicroclimOz repository, https://knb.ecoinformatics.org/view/doi:10.5063/F1833Q7X. WorldClim repository, https://www.worldclim.org/data/index.html.

## Code availability

All statistical analyses and output figures were produced using R statistical software (version 4.3.0)^48^. Code for producing temperature-dependent development outputs are available in Kearney 2019^34^ and associated supplementary material. Code used in this study to produce models is available at https://github.com/SciWithSteve/Species-distribution-GLM.
