# Supplementary material for: Establishing the distribution of Carpophilus truncatus in Australia using an integrative approach for an emerging global pest
Source: Sci Rep. 2024 Aug 22;14:19553. doi: 10.1038/s41598-024-70687-x (PMC11341852; doi:10.1038/s41598-024-70687-x)
Supplement: Supplementary file 5 — Supplementary Information 5. [file 41598_2024_70687_MOESM5_ESM.pdf]

Establishing the distribution of *Carpophilus truncatus* in Australia using an integrative approach for an emerging global pest

Stephen James Tobin, John Paul Cunningham

S5

Notes on methodology and results of statistical modelling

Geographic limitations of correlative SDM

The species distribution of *C. truncatus* in Victoria was predicted with a generalised linear model using presence-absence trap data. Modelling was possible in Victoria, where extensive trapping was performed over a range of environmental gradients[1,2]. While specimen records served to prove the presence of *C. truncatus* in the state of Queensland and possibly New South Wales and Western Australia, these data are not useful for distribution modelling: records indicate the species is present at a regional level, but the record data are too thin and lack accurate coordinates, and thus are not useful to meaningfully characterise the distribution of *C. truncatus* in their respective Australian states[3].

Differences in results between SDM and temperature-based model approaches

According to physiology-based predictions, much of Victoria could provide habitat where *C. truncatus* can complete multiple generations per year, but in contrast the GLM does not predict probability of presence in Victoria to follow along a consistent northwest-southeast gradient. There are several explanations that could be investigated. Habitat structure and the behavioural exploitation of microhabitats is likely to be important[4]. In almond orchards, *C. truncatus* spends most of its life feeding on the almond kernel within the almond shell, which is in turn encased in a leathery hull. This environment provides shelter for the beetle from predators and pesticides, and likely provides conditions that are much more stable than the general environment. If the insect follows similar habits in other locations, it may tolerate conditions that seem unfavourable at face value, and these processes may partially be captured by SDM but not by the temperature-development predictions. The 2.5cm MicroclimOz soil temperature layer used for temperature-based predictions may be less suitable in some geographic areas than others because the insects may adjust their pupation depth to optimise environmental conditions for development. Biological interactions may also partially explain the difference between the two approaches—although the insects could theoretically survive in areas solely according to temperature suitability, biotic factors such as lack of hosts, competition with other species, or predation likely affect their distribution in actuality[4–6]. Additionally, population dynamics, dispersal behaviour, as well as stochastic processes may affect when and where *C. truncatus* are lured into traps effectively and could go some way to explaining the moderate strength of model fit observed for the GLM[7,8]. Differences in catches between trapping seasons may reflect this, with moderate levels of presence predicted by the model in areas trapped during the first season, when trapping success was inconsistent at the regional level, compared to the second season when trapping success was consistent at regional level.

Rationale behind use of Generalized Linear Modelling, model performance metrics

The use of GLM over other modelling methods has a distinct advantage at a pragmatic level: most ecologists intuitively understand how a GLM receives input variables and produces predictions, unlike other unfamiliar, complex, or black-box modelling and machine learning methods[9]. Models are abstractions of reality, and while they are useful in providing specific

hypotheses to test, retaining interpretability should be encouraged through careful selection of SDM methods[1,10]. Future *C. truncatus* trapping would be beneficial to test the SDM presented in this study, as independent test data are typically a more effective for model validation than cross-validation and similar techniques, which have been proven to be unreliable indicators of model performance[8], especially in instances where the number of observations are small.

Model performance figures

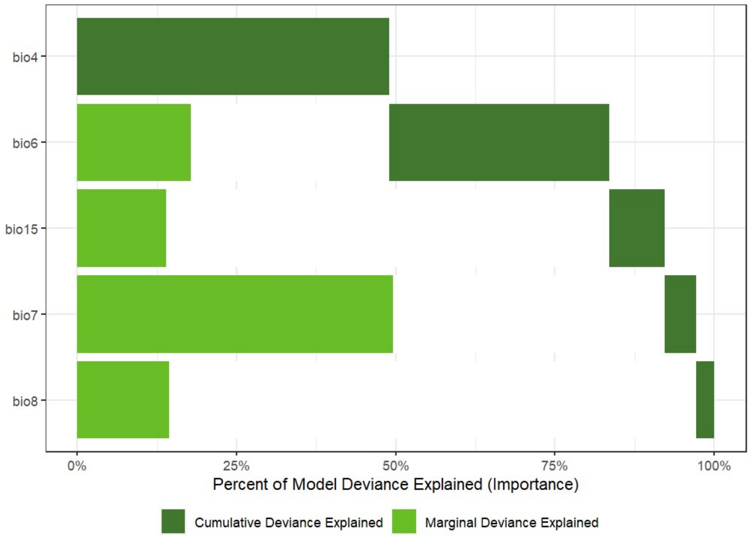

Supplementary Figure 1. Variable importance plot for the final GLM depicting the relative impact of each predictor variable on model fit, either alone or in the presence of other included predictor variables. Predictors are WorldClim bioclimate layers.

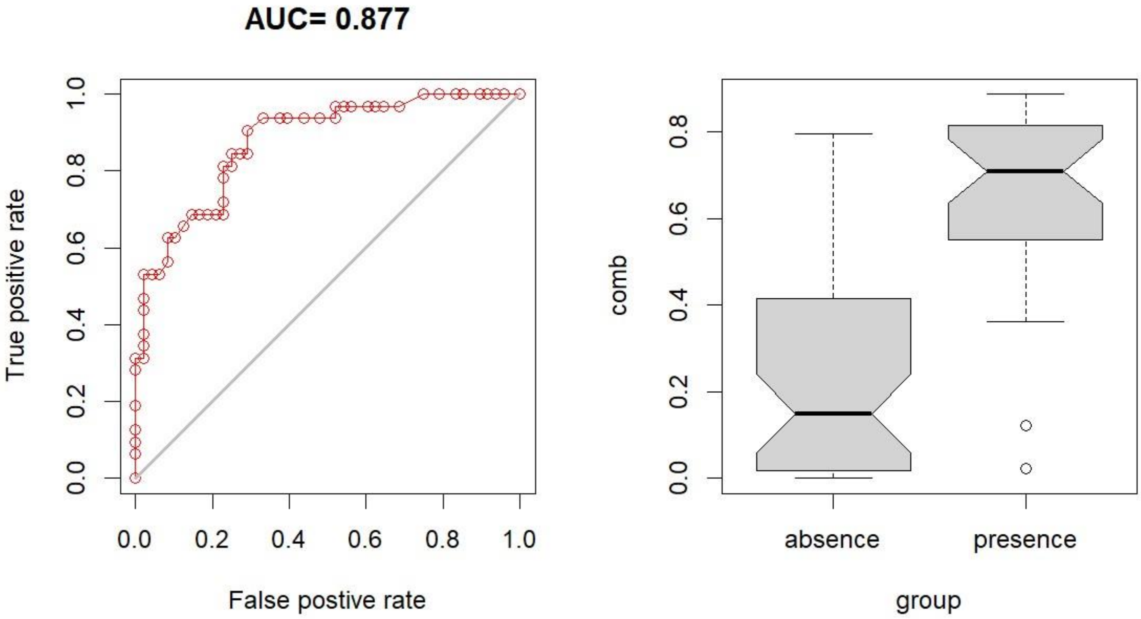

Supplementary Figure 2. Model performance metrics, receiver operating characteristic curve (ROC, left panel) and the distributions of model predicted values for presence and absence sites (right panel). The distributions of model predicted values for presence and absence sites shows the bulk of presences given predicted values higher than the bulk of absence values, but with some overlap, representing good but imperfect discrimination of presences and absences for this model.

References

1. Dormann, C. F. *et al.* Correlation and process in species distribution models: bridging a dichotomy. *J. Biogeogr.* **39**, 2119–2131 (2012).
2. El-Gabbas, A. & Dormann, C. F. Wrong, but useful: regional species distribution models may not be improved by range-wide data under biased sampling. *Ecol. Evol.* **8**, 2196–2206 (2018).
3. Papeş, M. & Gaubert, P. Modelling ecological niches from low numbers of occurrences: assessment of the conservation status of poorly known viverrids (Mammalia, Carnivora) across two continents. *Divers. Distrib.* **13**, 890–902 (2007).
4. Milling, C. R. *et al.* Habitat structure modifies microclimate: An approach for mapping fine-scale thermal refuge. *Methods Ecol. Evol.* **9**, 1648–1657 (2018).
5. Régnière, J., St-Amant, R. & Duval, P. Predicting insect distributions under climate change from physiological responses: spruce budworm as an example. *Biol. Invasions* **14**, 1571–1586 (2012).
6. Sillero, N. *et al.* Want to model a species niche? A step-by-step guideline on correlative ecological niche modelling. *Ecol. Model.* **456**, 109671 (2021).
7. Sutherst, R. W. Pest species distribution modelling: origins and lessons from history. *Biol. Invasions* **16**, 239–256 (2014).
8. A. Lee-Yaw, J., L. McCune, J., Pironon, S. & N. Sheth, S. Species distribution models rarely predict the biology of real populations. *Ecography* **2022**, e05877 (2022).
9. Jiménez-Valverde, A., Lobo, J. M. & Hortal, J. Not as good as they seem: the importance of concepts in species distribution modelling. *Divers. Distrib.* **14**, 885–890 (2008).
10. Maino, J. L., Kong, J. D., Hoffmann, A. A., Barton, M. G. & Kearney, M. R. Mechanistic models for predicting insect responses to climate change. *Curr. Opin. Insect Sci.* **17**, 81–86 (2016).
